# Expansion of rDNA and pericentromere satellite repeats in the genomes of bank voles *Myodes glareolus* exposed to environmental radionuclides

**DOI:** 10.1002/ece3.7684

**Published:** 2021-05-25

**Authors:** Toni Jernfors, John Danforth, Jenni Kesäniemi, Anton Lavrinienko, Eugene Tukalenko, Jiří Fajkus, Martina Dvořáčková, Tapio Mappes, Phillip C. Watts

**Affiliations:** ^1^ Department of Biological and Environmental Science University of Jyväskylä Jyväskylä Finland; ^2^ Department of Biochemistry & Molecular Biology Robson DNA Science Centre Arnie Charbonneau Cancer Institute Cumming School of Medicine University of Calgary Calgary Canada; ^3^ National Research Center for Radiation Medicine of the National Academy of Medical Science Kyiv Ukraine; ^4^ Mendel Centre for Plant Genomics and Proteomics Central European Institute of Technology (CEITEC) Masaryk University Brno Czech Republic; ^5^ Laboratory of Functional Genomics and Proteomics NCBR Faculty of Science Masaryk University Brno Czech Republic; ^6^ Department of Cell Biology and Radiobiology Institute of Biophysics of the Czech Academy of Sciences Brno Czech Republic

**Keywords:** anthropogenic disturbance, chernobyl, copy number, ionizing radiation, *myodes glareolus*, rDNA

## Abstract

Altered copy number of certain highly repetitive regions of the genome, such as satellite DNA within heterochromatin and ribosomal RNA loci (rDNA), is hypothesized to help safeguard the genome against damage derived from external stressors. We quantified copy number of the 18S rDNA and a pericentromeric satellite DNA (Msat‐160) in bank voles (*Myodes glareolus*) inhabiting the Chernobyl Exclusion Zone (CEZ), an area that is contaminated by radionuclides and where organisms are exposed to elevated levels of ionizing radiation. We found a significant increase in 18S rDNA and Msat‐160 content in the genomes of bank voles from contaminated locations within the CEZ compared with animals from uncontaminated locations. Moreover, 18S rDNA and Msat‐160 copy number were positively correlated in the genomes of bank voles from uncontaminated, but not in the genomes of animals inhabiting contaminated, areas. These results show the capacity for local‐scale geographic variation in genome architecture and are consistent with the genomic safeguard hypothesis. Disruption of cellular processes related to genomic stability appears to be a hallmark effect in bank voles inhabiting areas contaminated by radionuclides.

## Graphical abstract

Bank voles captured from locations contaminated with radionuclides in the Chernobyl Exclusion Zone exhibit increased copy numbers of ribosomal DNA and pericentromeric satellite Msat‐160, as well as a breakdown of genomic correlation between these loci compared with control. Copy‐number variation in heterochromatic repetitive loci may have adaptive significance.

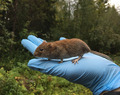

## 1 INTRODUCTION

**FIGURE 1:**
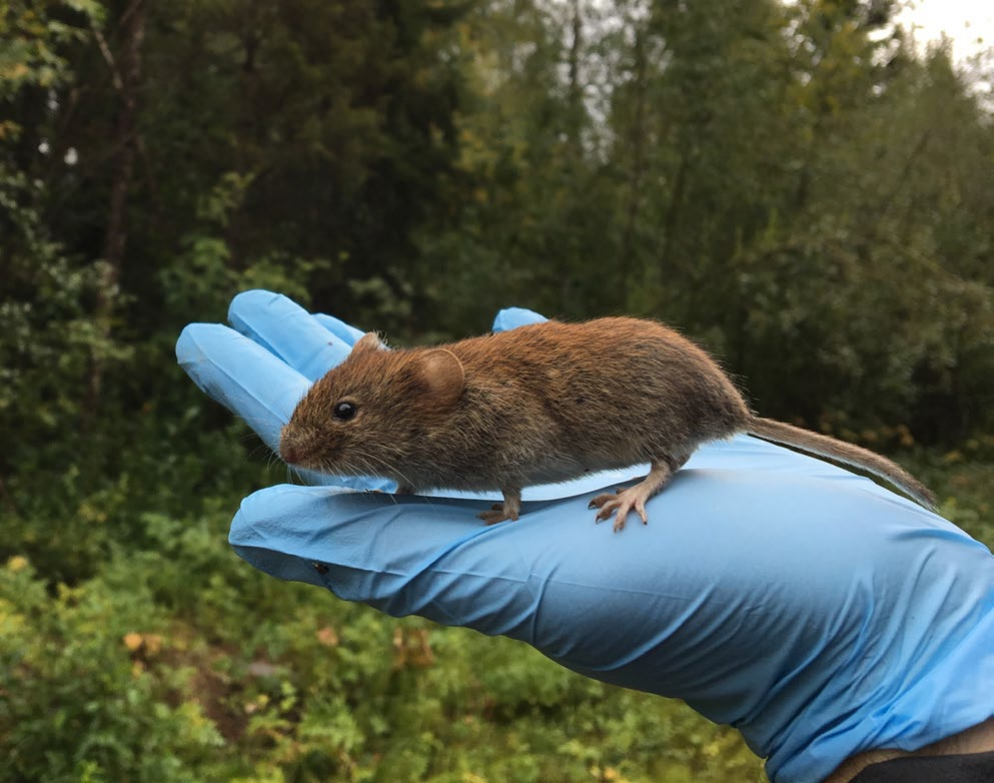
The bank vole *Myodes glareolus*

**FIGURE 2:**
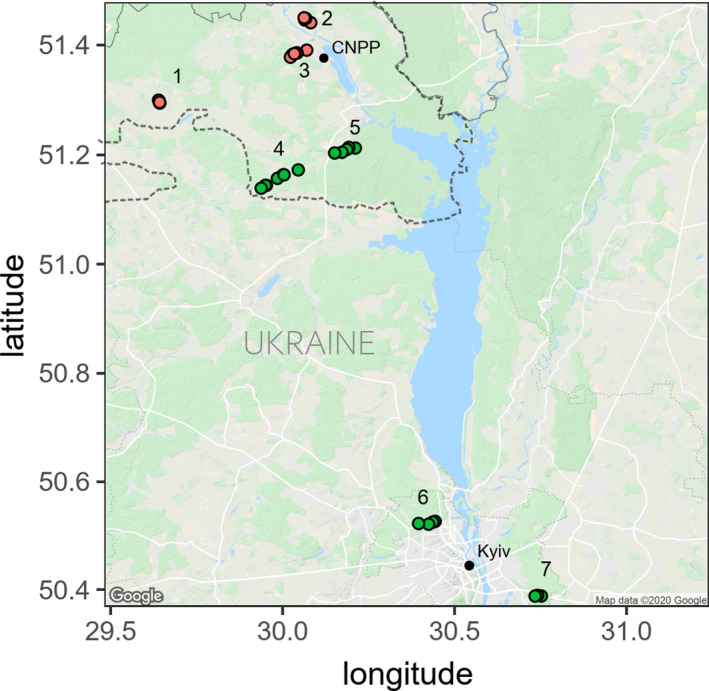
Location of *M. glareolus* sampling areas: contaminated CEZ (CEZ‐CNTM) areas (1) Vesnyane, (2) Gluboke, and (3) Chistogalovka; uncontaminated CEZ (CEZ‐CTRL) areas (4) Rosoha and (5) Yampil; and Kyiv (KYV‐CTRL) areas (6) Kyiv West and (7) Kyiv East. Dashed line represents the border around the CEZ in Ukraine (area of ~2,050 km^2^). Figure was created using ggmap v.3.0.0 package in R

Release of pollutants into the environment has diverse impacts upon wildlife, such as the bank vole (*Myodes glareolus*) (Figure 1) and the ecosystems they inhabit (Acevedo‐Whitehouse & Duffus, 2009; Isaksson, 2010). An example of environmental pollution whose potential impacts on wildlife have stimulated scientific debate is the fallout derived from the accident (April 26, 1986) at reactor 4 of the Chernobyl nuclear power plant, Ukraine, when approximately 9 million terabecquerels of radionuclides were released into the atmosphere and deposited across much of Eastern Europe, Russia, and Fenno‐Scandinavia (Beresford et al., 2016; Lourenço et al., 2016; Mousseau et al., 2014). The Chernobyl Exclusion Zone (CEZ) was established at an approximately 30‐km radius around the accident site (Figure 2) to limit human exposure to persistent radionuclides, notably strontium‐90, cesium‐137, and plutonium‐239 that have half‐lives of about 29, 30, and 24,100 years, respectively. In addition to controlled laboratory experiments, there is a need to study exposure to radionuclides in wildlife in natural habitats (Garnier‐Laplace et al., 2013), for which the CEZ provides a natural laboratory. Accordingly, the wildlife inhabiting the CEZ provide the best‐studied models of the biological impacts of exposure to environmental radionuclides (Beresford & Copplestone, 2011; Beresford et al., 2016; Bréchignac et al., 2016; Mappes et al., 2019; Mousseau et al., 2014).

Concern about the release of anthropogenic radionuclides into the environment stems from the potential damaging effects of exposure to ionizing radiation (IR) (Ward, 1988). For example, IR can damage DNA by direct impact that causes structural damage to DNA molecules, and/or by an indirect effect of radiolysis of cellular water that releases free radicals and causes an increase in oxidative stress (Desouky et al., 2015; Einor et al., 2016). Structural damage to DNA can induce, for example, genetic instability and abnormalities (such as cancers) or cell death. Elevated oxidative stress has diverse impacts on cell function, including an increase in DNA damage (Gonzalez‐Hunt et al., 2018; Poetsch et al., 2018). Indeed, many organisms inhabiting areas within the CEZ that are contaminated by radionuclides exhibit signs of elevated levels of genetic damage, such as increased frequency of chromosome aberrations (Dzyubenko & Gudkov, 2009) and/or an elevated mutation rate (Ellegren et al., 1997; Lourenço et al., 2016; Møller & Mousseau, 2015). Conversely, other studies have failed to find evidence for an increase in DNA damage (Rodgers & Baker, 2000), activation of DNA repair pathways (Kesäniemi, Jernfors, et al., 2019), or increase in mutation rate as measured by the level of heteroplasmy (Kesäniemi et al., 2018) in wildlife exposed to the persistent fallout from the Chernobyl accident. There are several possible reasons for the apparent support both for and against evidence of an increase in DNA damage in wildlife exposed to environmental radionuclides, such as interspecific variation in radiosensitivity (Beresford & Copplestone, 2011; Mousseau et al., 2014) and variation in received dose in the studied samples. Another key issue when quantifying DNA damage in wildlife exposed to environmental radionuclides is that certain regions of the genome preferentially accumulate damage when exposed to oxidative stress (Poetsch et al., 2018).

That regions of the genome differ in radiosensitivity is highlighted by evidence that an increase in frequency of four‐stranded G‐quadruplex (G4‐DNA) motifs can shield DNA against the direct damaging effects of IR in the human genome (Kumari et al., 2019). Also, radiosensitivity has been associated with telomeric DNA content in laboratory experiments on cell lines (Ayouaz et al., 2008; Zhang et al., 2016) and with minisatellite DNA in laboratory experiments on mice (Dubrova, 1998), although studies on the mutation rate at minisatellite loci in Chernobyl workers and their families have proven inconclusive (Bouffler et al., 2006). Interestingly, short telomeres characterized some tissues of bank voles inhabiting areas contaminated by radionuclides within the CEZ (Kesäniemi, Lavrinienko, et al., 2019), while slightly longer telomeres were found in blood of humans potentially exposed to high levels of radiation during the Chernobyl accident (Reste et al., 2014). It has been suggested that heterochromatin content can play a role in safeguarding transcribed genomic regions against the damaging effects of exposure to IR (Qiu, 2015).

Heterochromatin represents the transcriptionally suppressed, densely packed chromatin, which is mostly formed from repetitive non‐protein‐coding sequence (Grewal & Jia, 2007). The hypothesized role of heterochromatin in helping to safeguard the genome against IR‐induced damage (Qiu, 2015) is derived from the tendency for heterochromatin to localize at the nuclear periphery where it forms a three‐dimensional structure that physically surrounds the actively transcribed euchromatic regions (Geyer et al., 2011), possibly protecting these territories from impacts of IR and oxygen radicals. This “safeguard hypothesis” is supported by studies that demonstrate relationship between sensitivity to radiation, other mutagens or aging and loss in repetitive content such as telomeres (Goytisolo et al., 2000; Zhang et al., 2016), ribosomal DNA (rDNA) (Ide et al., 2010; Kobayashi, 2011, 2014), and heterochromatin in general (Larson et al., 2012; Yan et al., 2011). One paradox of the genomic safeguard hypothesis is that it should result in increased ratio of DNA damage in peripheral heterochromatin compared with euchromatin, contrary to observations where DNA repair activity is concentrated in the nuclear center than periphery (Gazave et al., 2005). However, damaged heterochromatic DNA is rarely repaired in situ (Chiolo et al., 2011), but is instead relocated to nuclear center for repair or to the nuclear pore complex to be expelled from the nucleus in the form of extrachromosomal circular DNAs (Chiolo et al., 2011; Jakob et al., 2011; Khadaroo et al., 2009; Qiu, 2015; Torres‐Rosell et al., 2007). Nonetheless, heterochromatin content of wild animals in the CEZ has not been explored. Two common constituents of heterochromatin, (a) rDNA and (b) centromeric DNA, are important for proper cellular function in eukaryotes and have been found to associate with genome stability (Kobayashi, 2011, 2014; Kobayashi & Sasaki, 2017). This possible association with genome stability implies that these genomic regions should be examined with regard to organisms inhabiting the CEZ.

The cluster of loci that are transcribed into ribosomal RNAs (hereafter called rDNA) represent a remarkable and evolutionarily conserved component of eukaryotic genomes. rDNA is organized as tandem arrays of 45S rRNA units that are transcribed and spliced into 18S, 5.8S, and 28S rRNAs, which, together with ribosomal proteins, form ribosomes. rDNA is abundant in many eukaryotic genomes, typically varying from tens to thousands of copies depending upon the species (Lavrinienko et al., 2020; Parks et al., 2018; Prokopowich et al., 2003; Symonová, 2019). Moreover, rDNA copy number can vary widely among individuals within a species (Lavrinienko, Jernfors, et al., 2020; Symonová, 2019; Weider et al., 2005), and geographic variation in rDNA content has been documented in diverse taxa (reviewed by Weider et al., 2005), for example, among plant populations that differ in altitude and latitude (Strauss & Tsai, 1988), in *Daphnia* (Harvey et al., 2020), and among human populations (Parks et al., 2018). As a tandemly repeating locus, rDNA is prone to copy‐number mutations, a feature that is exacerbated by topological stress from opening of the helical DNA structure and collisions between replication and transcription machinery due to frequent transcription (Salim & Gerton, 2019). Laboratory studies on fruit flies (*Drosophila melanogaster*) and baker's yeast (*Saccharomyces cerevisiae*) have demonstrated that exposure to stressors can elicit a rapid change in rDNA copy number within few generations (Aldrich & Maggert, 2015; Jack et al., 2015; Kobayashi, 2011; Paredes et al., 2011; Salim et al., 2017), making rDNA an apparently environmentally sensitive locus (Salim & Gerton, 2019). rDNA content is associated with genome stability and sensitivity to stress (Kobayashi & Sasaki, 2017). For example, strains of baker's yeast with fewer copies of rDNA are more sensitive to mutagens than strains with many rDNA copies (Ide et al., 2010). This interaction between rDNA and genome stability can be exemplified in plants, where dysfunction of chromatin assembly factor‐1 results in progressive loss of rDNA and higher sensitivity to genotoxic stress (Mozgová et al., 2010).

Another major fraction of heterochromatin is comprised of centromeric sequences and pericentromeric sequences, which flank the centromeres (Biscotti et al., 2015; Plohl et al., 2008). In contrast to rDNA, centromeric sequence motifs often are not evolutionarily conserved, but tend to differ among species (Biscotti et al., 2015). Centromeric DNA is defined by its ability to recruit the centromere‐specific histone 3 variant, centromere protein A (Foltz et al., 2006). Centromere and pericentromere regions are typically comprised of tandem arrays of satellite DNA such as α‐satellites in primates (Alexandrov et al., 2001), minor and major satellites in mice (Komissarov et al., 2011), and an approximately 160‐base‐pair‐long satellite motif (Msat‐160) in arvicoline rodents (Acosta et al., 2010). Using fluorescence in situ hybridisation (FISH), Msat‐160 has often been characterized as an abundant component of arvicoline rodent genomes, located primarily at the pericentromeric regions of chromosomes and with apparently high interspecific variation in copy number and the number of chromosomes that contain the satellite sequence (Acosta et al., 2007, 2010; Modi, 1992, 1993). While interspecific differences in centromere architecture are quite well described for some taxa (*e.g*., primates, Melters et al., 2013), perhaps consistent with the general lack of information about centromere and pericentromere sequence in most species, we are not aware of any studies to have quantified whether variation in pericentromeric satellite content associates with features of the environment. However, as constituents of heterochromatin, the centromeric and pericentromeric regions may represent a key component of the genome that interacts with exposure to environmental stress.

Bank voles appear to be relatively radioresistant, being one of the first mammals to recolonize the Chernobyl accident site (Chesser et al., 2000) and with animals exposed to environmental radionuclides showing equivocal evidence for genomic DNA damage (Rodgers & Baker, 2000), little upregulation of DNA repair pathways (Jernfors et al., 2018; Kesäniemi, Jernfors, et al., 2019), and no elevated mutation rate (heteroplasmy) in their mitochondrial genomes (Kesäniemi et al., 2018). At a cellular level, fibroblasts isolated from bank voles exposed to radionuclides in the CEZ show increased resistance to oxidative stress and genotoxins (Mustonen et al., 2018). Given the genomic safeguard hypothesis, we expect that change in rDNA and pericentromere content will be a feature of the genomes of organisms exposed to radionuclides. To test this hypothesis, we used quantitative PCR (qPCR) to quantify the relative amounts of (a) 18S rDNA and (b) Msat‐160 (a pericentromeric satellite sequence) as proxies for heterochromatin content in genomes of bank voles that have inhabited areas contaminated with radionuclides for estimated 50 generations (Baker et al., 2017).

## 2 MATERIALS AND METHODS

### 2.1 Sampling and dosimetry

Two hundred and two bank voles were captured using Ugglan Special2 live traps with sunflower seeds and potatoes as bait during fieldwork seasons of 2016–2017. Briefly, at each location 9–16 traps were placed either in a 3 × 3 or 4 × 4 grid with an intertrap distance of 15–20 m. In all locations, traps were kept for at least three consecutive nights and were checked each following morning. Animals were brought to a field laboratory in town of Chernobyl within the CEZ where they were euthanized by cervical dislocation within 24 hr of entering the laboratory and stored in dry ice for transport before long‐term storage in −80°C. rDNA copy number can change within a generation (Aldrich & Maggert, 2015); in bank voles, stress during early (but not adult) life can affect rDNA copy number (van Cann, 2019). Animals were caught from seven study areas in Ukraine (Figure 2), with near‐equal sex ratios at each location (Rosoha: 14 females/14 males, Yampil: 15F/15M, Chistogalovka: 15F/15M, Gluboke: 15F/14M, Vesnyane: 15F/15M, East Kyiv: 15F/12M, and West Kyiv: 15F/13M). We measured ambient radiation levels at all trapping locations using a handheld Geiger counter (Gamma‐Scout GmbH & Co.) placed 1 cm above the ground, and taking an average of at least nine measurements from each trapping location. Ambient dose rate measurements provide a reasonable approximation of external absorbed dose rate for bank voles (Beresford et al., 2008; Lavrinienko, Tukalenko, et al., 2020). Internal absorbed cesium‐137 dose rates were measured using a SAM 940 radionuclide identifier system (Berkeley Nucleonics Corporation). Full details on internal dosimetry and total received dose rate estimations are provided in Appendix 1 and supporting data.

**TABLE 1:** Sequences of qPCR primers that amplify fragments of the 18S rDNA and Msat‐160 (and 36b4 as a single‐copy control gene) in the bank vole *Myodes glareolus*

locus		Sequence (5′→3′)	Amplicon length (bp)
18S rDNA	F	AAG ACG GAC CAG AGC GAA AG	238
	R	TGG TGC CCT TCC GTC AAT TC	
Msat‐160	F	CAG CAT TTA GAA AGT GAA GCA ACA	101
	R	CCA AGA AAC TCA CAG GCA TTT C	
36b4	F	GTC CCG TGT GAA GTC ACT GT	87
	R	AGC GGT GTT GTC TAA AGC CT	

To control for possible confounding variables, we use a robust study design that utilized samples from replicated contaminated areas within the CEZ and noncontaminated areas within and outside the CEZ. We classified our seven study areas into three exposure groups that reflect a likely radiation “treatment.” Three areas within the CEZ (Chistogalovka, Gluboke, and Vesnyane) were contaminated by radionuclides and delivered elevated external dose rates (0.24–1.49 mGy/day, median 0.41 mGy/day, representing ~4 chest X‐rays per day) to wildlife inhabiting these areas: These three sites are collectively referred to as CEZ‐CNTM (CEZ‐contaminated). Two areas within the CEZ (Rosoha and Yampil) had little to no apparent soil radionuclide contamination, with near‐background external dose rates (median 6.4*10^–3^ mGy/day), and are referred to as CEZ‐CTRL (CEZ‐control). Finally, the two areas outside the CEZ (Kyiv East and Kyiv West) also are uncontaminated by environmental radionuclides (median 3.6*10^–3^ mGy/day) and are referred to as KYV‐CTRL (KYIV‐control). As the CEZ presents a mosaic of radionuclide contamination (Mousseau et al., 2014), we make the distinction between the locations defined as CEZ‐CTRL and KYV‐CTRL. At CEZ‐CTRL, it is possible that some animals caught in these uncontaminated areas may have dispersed into these areas from contaminated areas (and indeed vice versa). By contrast, KYV‐CTRL presents a sample of animals that have not directly encountered a large dose of IR from environmental radionuclides, because the distance between the KYV‐CTRL site and the CEZ (~90 km) is much further than bank voles’ dispersal ability (*ca*. ~1 km per breeding season, Kozakiewicz et al., 2007; and an estimated <5 km per year rate of range expansion, White et al., 2012; Smiddy et al., 2016).

### 2.2 Characterization of bank vole 18S rDNA and Msat‐160 satellite sequences and primer design

Sequences for ribosomal rDNA were identified by BLASTn search (Altschul et al., 1990; parameters: default) of mouse 18S rDNA (GenBank Accession NR_003278.3) against a draft bank vole genome (GenBank Accession GCA_001305785.1). The putative bank vole pericentromere satellite DNA sequence was identified by its similarity (70% identity, *e*‐value = 3e^−14^) with the satellite DNA Msat‐160, clone 960‐47 isolated from the genome of the Eurasian water vole *Arvicola amphibius* (synonym *A. terrestris*) (GenBank Accession FN859393.1). Quantitative PCR (qPCR) primers for 18S rDNA and Msat‐160 sequences were designed using Primer3web v.4.1.0 (Untergasser et al., 2012) and BLAST to ascertain primer specificity.

### 2.3 Fluorescence in situ hybridization of Msat‐160 satellite in the bank vole genome

Arvicoline rodents display high variability in centromere sequence composition owing to rapid species radiation (Acosta et al., 2010). To identify genomic locations of the Msat‐160 satellite sequence in the bank vole genome, fluorescence in situ hybridization (FISH) was used using the same fibroblast source as Mustonen et al. (2018). Fibroblasts were isolated from male bank voles collected from Gluboke (Figure 2) and from near Kyiv, cultured according to Mustonen et al. (2018), and fixed in 3:1 methanol:glacial acetic acid according to the standard protocol (Franek et al., 2015). Full details of chromosomal preparations are given in Appendix 2. FISH images were taken using Zeiss Axio Imager Z2 microscope, using a Plan‐Apochromat 100×/1.4 OIL objective and an ORCA Flash 4 camera. Images were analyzed with CellProfiler 2.4.

### 2.4 Quantitative PCR to detect relative copy number of 18S rDNA and Msat‐160

Genomic DNA was extracted from ear tissue samples using DNeasy Blood & Tissue Kit (Qiagen) following the manufacturer's protocol. Quantitative PCRs were carried out on a LightCycler 480 Real‐Time PCR System (Roche), using ribosomal phosphoprotein P0‐coding gene *36b4* as a single‐copy reference gene (see Cawthon, 2002). Each 96‐well plate of qPCRs contained triplicate reactions for the same reference bank vole DNA sample that acted as a golden standard (GS). Raw quantification cycle (Cq) data were corrected for PCR efficiency (E) and transformed into relative values compared with a single‐copy reference using the Pfaffl method (Pfaffl, 2001), where

$$\text{ratio}=\frac{{\left(\text{Etarget}\right)}^{\Delta \text{Cqtarget}(\text{control}‐\text{sample})}}{{\left(\text{Eref}\right)}^{\Delta \text{Cqref}(\text{control}‐\text{sample})}}.$$

All qPCRs were run in 15 μl final reaction volumes using LightCycler 580 SYBR Green I Master (Roche). Reactions for Msat‐160 and *36b4* included 15 ng template DNA, 500 nM of both forward and reverse primers for Msat‐160, and 200 nM of both primers for *36b4*. Reactions for 18S rDNA included 3 ng genomic DNA and 333 nM of both forward and reverse primers. Amplification conditions for Msat‐160 were as follows: denaturation at 95°C for 5 min, and 40 cycles of amplification (95°C for 10 s, 58°C for 5 s, and 72°C for 5 s). Amplification conditions for *36b4* were as follows: 95°C 5 min and 40 × (95°C 10 s; 58°C 15 s; 72°C 10 s). Amplification conditions for 18S were as follows: 95°C and 45 × (95°C 10 s; 60°C 15 s; 72°C 10 s). A standard melt curve analysis included in the LightCycler software was included in each analysis run to ascertain product specificity. All samples whose duplicate *C*_t_ values had standard deviations above 0.2 cycles were rerun. Dilution series of GS DNA were run on each qPCR plate to generate PCR efficiencies: For Msat‐160, we used a 1:5 dilution series from 40 to 0.064 ng/μl; for *36b4*, we used a 1:3 dilution series from 40 to 0.49 ng/μl; and for 18S, we used a 1:5 dilution series from 12.0 ng to 0.0192 ng/µl.

Variation in relative copy number for 18s rDNA and for Msat‐160 (response variables) was evaluated using linear mixed models in lme4 (Bates et al., 2015) in R v.3.5.0 (The R Core Team, 2018), including radiation treatment group (CEZ‐CNTM, CEZ‐CTRL, and KYV‐CTRL) and sex as fixed factors and trap point (*N* = 56) or trapping area (*N* = 7) as a random factor. Marginal $R_{m}^{2}$ (variance explained by fixed effects) and conditional $R_{c}^{2}$ (variance derived from fixed and random effects), as well as the significance of post hoc comparisons, were calculated using MuMIn (Burnham & Anderson, 2002) and multcomp (Hothorn et al., 2008), also in R. We also ran models that examined the total received dose rates as a continuous variable (instead of treatment).

## 3 RESULTS

**FIGURE 3:**
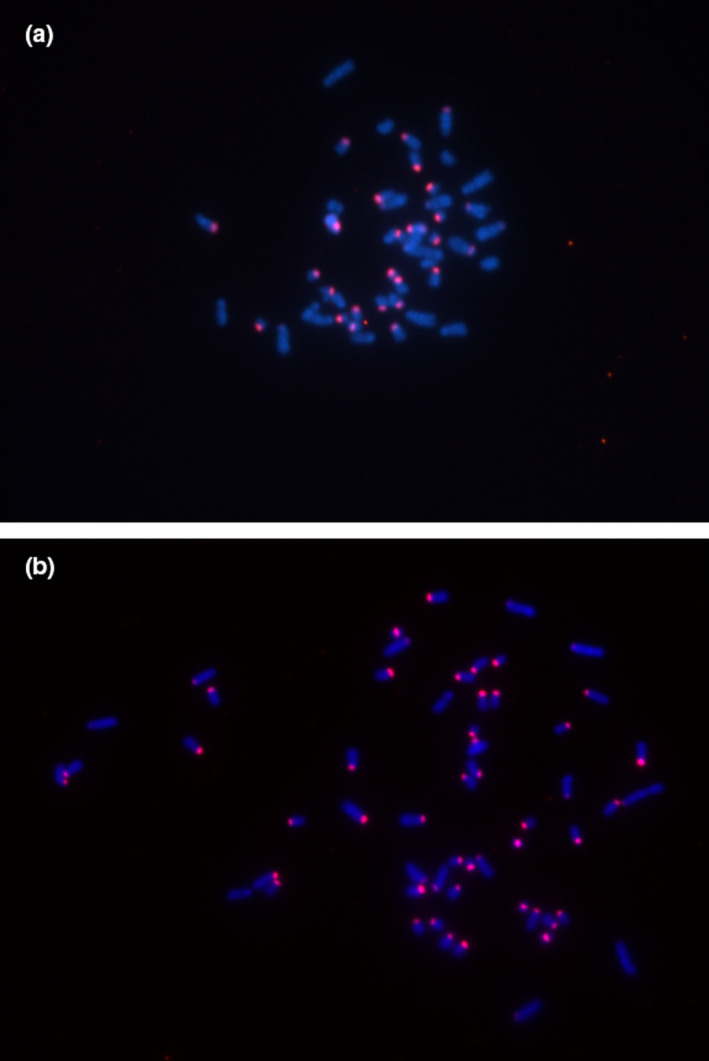
Fluorescence in situ hybridization (FISH) staining of Msat‐160 regions in *M. glareolus* fibroblast cells (red). (a) Cells from a male individual sampled from the Kyiv West site and (b) cells from a male individual from CEZ‐CNTM Gluboke site. Chromosomes were counterstained with DAPI (blue)

FISH confirmed that Msat‐160 is located in the pericentromeric heterochromatin regions in the bank vole genome. Most bank vole chromosomes are acrocentric (one‐armed), a feature that is common among rodents (Pardo‐Manuel de Villena & Sapienza, 2001) (Figure 3).

**FIGURE 4:**
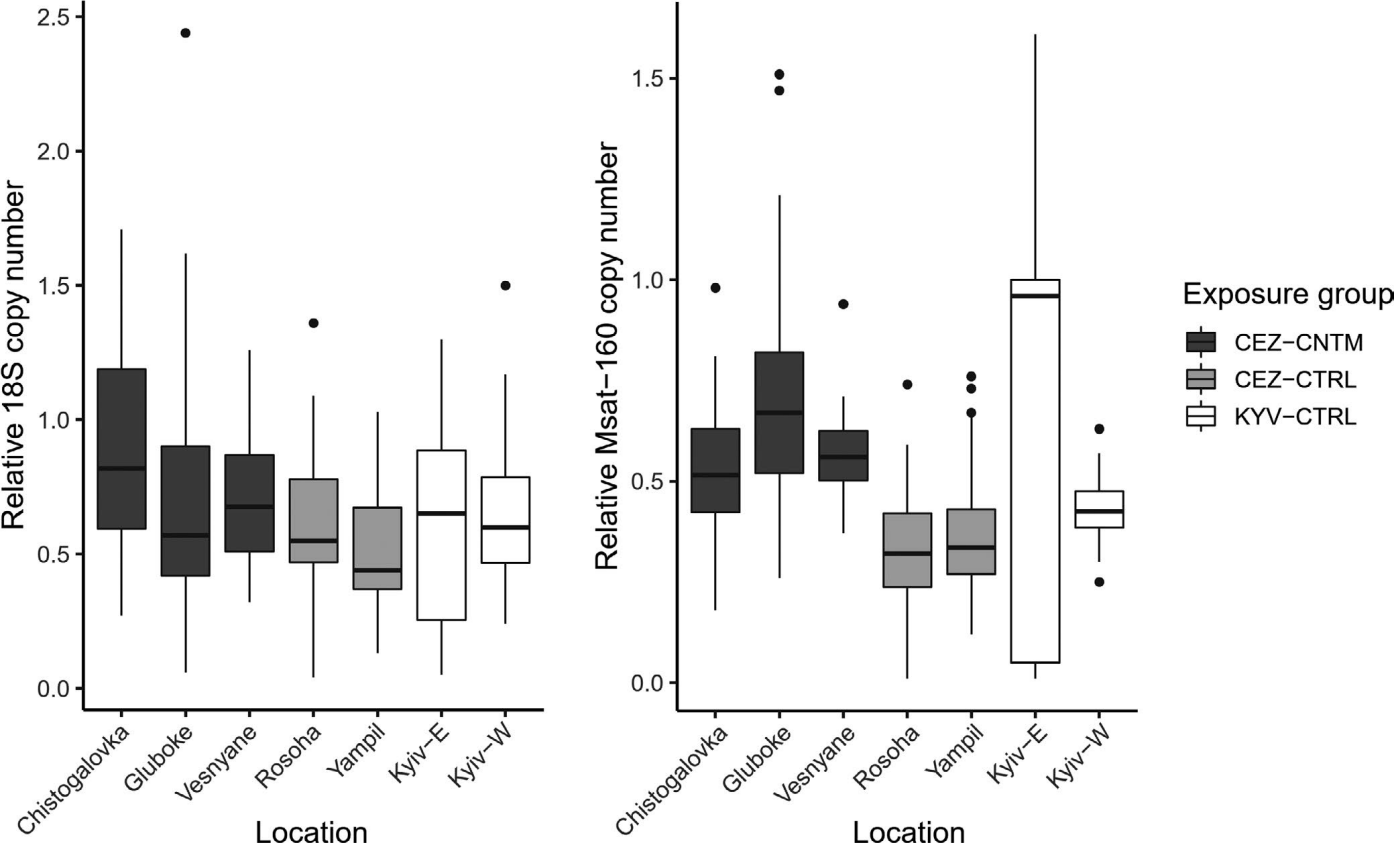
Genomic changes in response to exposure to ionizing radiation as measured by (a) relative 18S rDNA and (b) Msat‐160 copy number in bank voles inhabiting areas contaminated by radionuclides (CEZ‐CNTM) and uncontaminated areas (CEZ‐CTRL and KYV‐CTRL). Relative copy numbers of both repeats are normalized to a reference golden standard DNA sample. Dots represent data points above 1.5 * interquartile range

We observed significant differences in the relative copy number of 18S rDNA in bank voles among the three radiation treatments ($R_{m}^{2}$ = 0.128, $R_{c}^{2}$ = 0.137, Table 2, Figure 4a). Consistent with the genomic safeguard hypothesis, average rDNA content was significantly higher in animals caught from CEZ‐CNTM compared with animals from CEZ‐CTRL areas (β = 0.195, *df* = 7.12, *t* = 3.15, *p* < 0.05), although then comparison between CEZ‐CNTM and KYV‐CTRL was not significantly different. Male bank voles had significantly more copies of 18S rDNA than females (β = 0.182, *df* = 195.34, *t* = 4.01*, p* < 0.001), except in the sample from East Kyiv.

**TABLE 2:** Primary models explaining variation in mean relative copy number of ribosomal 18S DNA and centromeric repeat Msat‐160 as dependent variables (DV) between treatment groups (Ex.grp) using CEZ‐CTRL and female voles as reference level

DV (fixed effects)		β (SE)	*df*	*t*
18S copy number	Intercept	0.483 (0.053)	10.762	9.087***
(Ex. Grp+Sex)^a^	CEZ‐CNTM	0.195 (0.062)	7.120	3.153*
	CTRL‐KYV	0.073 (0.069)	7.540	1.057
	Sex (Male)	0.182 (0.045)	195.337	4.008***
Msat−160 copy number	Intercept	0.352 (0.044)	77.86	7.968***
(Ex. Grp+Sex)^b^	CEZ‐CNTM	0.251 (0.051)	68.26	4.949***
	CTRL‐KYV	0.174 (0.064)	32.55	2.725*
	Sex (Male)	0.007 (0.038)	197.7	0.180

^a^ Random factor: trapping area, *N* = 7, Var = 0.001 (trapping point resulted in singular fit).

^b^ Random factor: trapping point, *N* = 56, Var = 0.005.

* *p* < 0.05

*** *p* < 0.001.

Msat‐160 satellite motif (pericentromere) also exhibited significant spatial variation in copy number among treatment groups ($R_{m}^{2}$ = 0.135, $R_{c}^{2}$ = 0.192, Table 2, Figure 4b). Again, consistent with the genome safeguard hypothesis, bank voles caught from contaminated areas within the CEZ had significantly more copies of Msat‐160 than did animals caught from uncontaminated areas within the CEZ (β = 0.251, *df* = 68.26, *t* = 4.949, *p* < 0.001). Mean copy number in KYV‐CTRL was significantly higher than in CEZ‐CTRL (β = 0.174, *df* = 32.55, *t* = 2.725, *p* < 0.05). Male voles have higher Msat‐160 content than females in all areas except at East Kyiv, where females have elevated Msat‐160 content, thus increasing the average Msat‐160 content in the KYV‐CTRL sample.

Both 18S rDNA and Msat‐160 copy number also significantly correlated with radiation levels when modeling total absorbed dose rate as a continuous variable (β = 0.020, *df* = 7.26, *t* = 0.042, *p* < 0.05 and β = 0.028, *df* = 70.38, *t* = 3.985, *p* < 0.001, respectively) (Table 3). When considering bank voles only within the CEZ‐CNTM group (as other areas had near‐zero radiation levels), neither 18S rDNA nor Msat‐160 copy number correlated with the total received dose rate (*R* = 0.02 and 0.11, respectively, for log dose, *p* > 0.05).

**TABLE 3:** Alternative models for Msat‐160 and 18S rDNA content using logarithm of the total received dose rate (D.rate, mGy/day) as a continuous variable in place of treatment group‐based models. Female voles are used as reference level

DV (fixed effects)		β (SE)	*df*	*t*
18S copy number	Intercept	0.676 (0.050)	10.75	13.461***
(D.rate+Sex)^a^	D.rate	0.020 (0.008)	7.26	0.042*
	Sex (Male)	0.180 (0.046)	195.20	3.939***
Msat−160 copy number	Intercept	0.614 (0.041)	118.90	14.995***
(D.rate+Sex)^b^	D.rate	0.028 (0.007)	70.38	3.985***
	Sex (Male)	0.010 (0.039)	201.70	0.267

^a^ Random factor: trapping area, *N* = 7, Var = 0.002 (trapping point resulted in singular fit).

^b^ Random factor: trapping point, *N* = 56, Var = 0.009.

* *p* < 0.05

*** *p* < 0.001.

**FIGURE 5:**
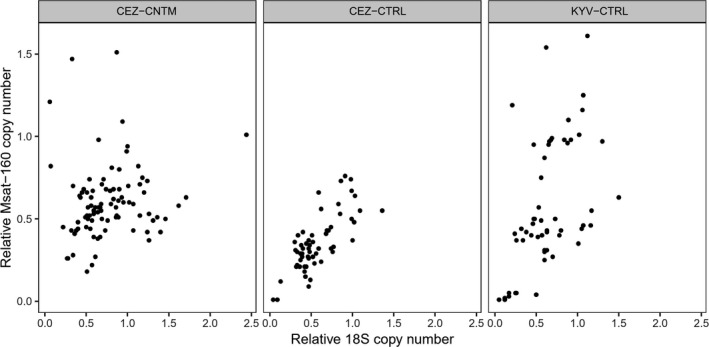
Association between 18S rDNA and Msat‐160 content. 18S rDNA and Msat‐160 content are correlated in all uncontaminated areas (CEZ‐CTRL and KYV‐CTRL), but not in contaminated areas (CEZ‐CNTM)

Given the similar increase in 18S rDNA and Msat‐160 content in the genomes of animals exposed to environmental radionuclides, we quantified (a) whether the copy number at these loci was correlated within individuals (*i.e*., is there an intragenomic correlation in rDNA and Msat‐160 content) and (b) whether the strength of any such intragenomic correlations was impacted by exposure to environmental radionuclides. Significant positive correlations were found (Pearson's correlation, *R* > 0.5 and *p* < 0.05, with weaker correlation in the West Kyiv sample) between the copy number of 18S rDNA and Msat‐160 in animals at all uncontaminated areas (Table 4, Figure 5). However, this intragenomic correlation in copy number among 18S rDNA and Msat‐160 was not observed (*R* < 0.3, *p* > 0.1) at the contaminated areas (CEZ‐CNTM).

**TABLE 4:** Pearson's correlation between 18S rDNA and Msat‐160 copy numbers among treatment groups and trapping areas

Exposure group	Trapping area	*R*	*df*	*t*
CEZ‐CNTM	Combined	0.130	87	1.213
	Gluboke	0.165	27	0.868
	Vesnyane	0.251	28	1.374
	Chistogalovka	0.279	28	1.540
CEZ‐CTRL	Combined	0.755	56	8.618***
	Yampil	0.831	28	7.890***
	Rosoha	0.808	26	7.005***
KYV‐CTRL	Combined	0.515	53	4.370***
	East	0.724	25	5.540***
	West	0.387	26	2.141*

* *p* < 0.05

*** *p* < 0.001.

## 4 DISCUSSION

### 4.1 Increased genomic repeat copy number may mitigate radiation stress

It is hypothesized that heterochromatin may uphold genomic stability by safeguarding the genome against environmental stresses such as ionizing radiation (Qiu, 2015). We found (a) that both 18S rDNA and Msat‐160 satellite (both of which are major constituents of heterochromatin) copy numbers were higher in contaminated areas than uncontaminated areas within the CEZ, (b) extensive variation in copy number at KYV‐CTRL, and also (c) intragenomic correlations in rDNA and Msat‐160 content at CEZ‐CTRL and at KYV‐CTRL, but not in the samples from the contaminated areas (CEZ‐CNTM).

An apparent increase in both Msat‐160 and rDNA copy number in contaminated sites is consistent with the genomic safeguard hypothesis (Qiu, 2015). Loss of heterochromatin as an expectation of the hypothesis is mainly a result of relocation and expulsion of DNA double‐strand breaks that we do not expect in the CEZ due to the low‐dose rates generally thought to be too weak to induce them. Increased heterochromatin content could provide stability against oxidative stress, and thus, changes in rDNA and Msat‐160 content, either by selection on existing copy‐number variation or by affecting copy‐number maintenance during development, may be an adaptive response to exposure to environmental radionuclides. Interestingly, our data show how this change in genome architecture can occur at a local geographic scale (*i.e*., within a few tens of km). Considering animals within contaminated areas (CEZ‐CNTM) only, direct effect of IR on heterochromatin content is not expected, for example, because we do not expect DNA content to have linear response to IR, and dose measured may not reflect that experienced at the relevant time of life, that is, embryonic development.

rDNA copy‐number variation has been associated with diverse cellular functions (Gibbons et al., 2014; Kobayashi & Sasaki, 2017), and loss of rDNA copies correlates with, for example, cellular senescence and susceptibility to mutagens in yeast (Ide et al., 2010; Kobayashi, 2011). Aside from possible safeguarding the genome as part of heterochromatin, changes in rDNA content can affect cell processes such as transcription (Gibbons et al., 2014; Jack et al., 2015; Paredes et al., 2011; Parks et al., 2018; Salim et al., 2017) and nucleolus organization (Potapova & Gerton, 2019). Loss of rDNA in mouse cancer lines is associated with vulnerability to DNA damage and activation of the mTOR pathway, which promotes cell growth and division (Xu et al., 2017). Interestingly, mTOR activation is antagonistic to a fatty acid oxidating mode of metabolism. Bank voles inhabiting contaminated areas within the CEZ upregulate genes associated with fatty acid oxidation (Kesäniemi, Jernfors, et al., 2019), which is associated with genomic stability (Heydari et al., 2007; Yuan et al., 2013) in the form of antioxidative capabilities and longevity in captivity. While the explicit link between rDNA CN variation and metabolic changes in the bank vole remains unclear, fibroblasts isolated from bank voles exposed to radionuclides show increased tolerance of oxidative stress and genotoxic agents (Mustonen et al., 2018). Increase in rDNA copy number may thus provide certain advantages to bank voles exposed to environmental radionuclides, although this idea remains to be tested experimentally.

Like a change in rDNA copy number, change in pericentromeric content associated with environmental radionuclides is interesting as this change in genome architecture can have diverse impacts on cell function. For example, centromeric and pericentromeric regions contain noncoding RNA (ncRNA) sequences that are involved in centromere maintenance and gene silencing (Ideue & Tani, 2020). Altered centromere structure can change patterns of histone binding with a concomitant impact on gene expression (Vaissière et al., 2008). Also, given the shorter telomeres in bank voles exposed to radionuclides (Kesäniemi, Lavrinienko, et al., 2019), it is interesting that many bank vole chromosomes are acrocentric (Figure 3) as this possibly makes Msat‐160 simultaneously pericentromeric and subtelomeric. Subtelomeric heterochromatin can contribute to telomere protection, for example, by inducing compaction of telomere chromatin to a less accessible chromatin structure or even replacing canonical telomeres in telomerase‐independent pathways of telomere maintenance (Jain et al., 2010). Subtelomeric repeats also show high copy‐number variation under some stress conditions with detrimental consequences (Chow et al., 2012; van der Maarel & Frants, 2005; Vyskot et al., 1991).

It is perhaps important to note that, even with an attempt at genomic safeguard to mitigate some of the genomic consequences of stress, animals inhabiting contaminated areas can still experience diverse impacts of exposure to radionuclides. For example, bank voles inhabiting contaminated areas within the CEZ show reduced breeding success (Mappes et al., 2019) and elevated frequency of cataracts (Lehmann et al., 2016). Also, bank voles exposed to radionuclides show an increase in damage to their mitochondrial genomes (Kesäniemi et al., 2020), possibly because the mitochondria do not use certain DNA repair pathways (*e.g*., nucleotide excision repair) and/or the mitochondrial DNA lacks heterochromatin (Kazak et al., 2012; Yakes & Van Houten, 1997).

### 4.2 Breakdown of intragenomic correlations as a hallmark of radioactively contaminated habitat

The correlation between rDNA and Msat‐160 copy number in bank voles from uncontaminated areas within and outside the CEZ, but not in animals from contaminated areas, implies some disruption to typical genome architecture when animals are exposed to environmental radionuclides. Repetitive genome fraction generally correlates with genome size between species (Gregory, 2001; Prokopowich et al., 2003). Data on expected intraspecific correlations among different genomic regions are lacking, although there is some evidence that rDNA content negatively associates with mitochondrial DNA content in humans (Gibbons et al., 2014). We are not aware of any previous report that rDNA and pericentromere content would be correlated. Regardless, disruption to processes that maintain typical cell and genome integrity is a notable feature of bank voles inhabiting areas contaminated by radionuclides, such as (a) a lack of correlation in telomere length among different tissues (Kesäniemi, Lavrinienko, et al., 2019), (b) no relationship between mitochondrial DNA copy number and expression of PGC1α (the gene that regulates mitochondrial synthesis) in brain tissue (Kesäniemi et al., 2020), and (c) a weakening of gene coexpression networks in liver and spleen (Kesäniemi, Jernfors, et al., 2019).

### 4.3 Safeguard by genomic repeats may respond to diverse stressors

The comparison between samples from the CEZ and West Kyiv is consistent with the genomic safeguard hypothesis, but the general increase in rDNA and Msat‐160 copy number in the genomes of animals from East Kyiv cannot be explained. On the one hand, these data could be used to argue against an increase in rDNA and centromeric DNA to exposure to IR. However, as diverse stressors can elicit intraspecific heterogeneity in rDNA content (Govindaraju & Cullis, 1992; Harvey et al., 2020; Salim et al., 2017), and potentially centromeric architecture, it is possible that voles at East Kyiv experienced some other feature of the environment that selected for an increase in rDNA/centromere content. As such, the data from East Kyiv do not necessarily refute the idea of genome safeguard by heterochromatin. Also, we are not aware of other reports of sex differences in rDNA content in mammals. In *Drosophila melanogaster*, the rDNA cluster is located on sex chromosomes. However, the rDNA clusters in mammals, such as humans and mice, are located on several autosomes (Coen & Dover, 1983) and not sex chromosomes. In any case, an animal's sex and its rDNA content do not interact with radionuclide exposure. Hence, our data highlight the capacity for wild animals to show macro‐ and microgeographic variation in DNA and Msat‐160 (*i.e*., centromeric) content, and this emphasizes the need for further research to understand the biological driver(s) of this genetic variation. Particularly important will be the use of experiments to quantify how specific stressors, such as environmental radionuclide exposure, impact these apparently labile regions of the genome. Moreover, heterochromatin architecture as a potential mitigator of genome damage should be explored in more detail, not only in bank voles, but also in other species that differ in apparent radiosensitivity (Lourenço et al., 2016) and amount of noncoding DNA (Gregory, 2001; Prokopowich et al., 2003).

## 5 CONCLUSION

In conclusion, we uncovered geographic variation in Msat‐160 and 18S rDNA content in bank vole genomes that is consistent with the hypothesized role of heterochromatin and rDNA in responding to, and safeguarding the genome against, environmental stress. Furthermore, we show loss of an apparent intragenomic correlation in rDNA‐(peri‐)centromere content in bank voles exposed to radionuclides. While the role of non‐protein‐coding DNA, and particularly rDNA, is well researched in a medical context (Kobayashi, 2014; Wang & Lemos, 2017; Xu et al., 2017), our data indicate clear potential for environmental pollution to have a broad effect in genome architecture. Additional studies are required to partition the plastic and heritable components of these copy‐number changes, and also whether this change in genome architecture is part of an adaptive response to exposure to elevated radiation dose or, indeed, other pollutants.

## CONFLICT OF INTEREST

The authors declare no competing interests.

## AUTHOR CONTRIBUTION

**Toni Jernfors:** Conceptualization (equal); Formal analysis (lead); Investigation (equal); Writing‐original draft (lead). **John Danforth:** Conceptualization (equal); Investigation (equal); Writing‐review & editing (equal). **Jenni Kesäniemi:** Investigation (equal); Writing‐review & editing (equal). **Anton Lavrinienko:** Investigation (equal); Resources (equal); Writing‐review & editing (equal). **Eugene Tukalenko:** Investigation (equal); Resources (equal); Writing‐review & editing (equal). **Jiří Fajkus:** Resources (equal); Writing‐review & editing (equal). **Martina Dvořáčková:** Investigation (equal); Writing‐review & editing (equal). **Tapio Mappes:** Resources (equal); Writing‐review & editing (equal). **Phillip Watts:** Conceptualization (lead); Project administration (lead); Supervision (lead); Writing‐review & editing (lead).

## ETHICAL APPROVAL

All experiments complied with the legal requirements and adhered closely to international guidelines for the use of animals in research. All necessary permissions were obtained from the Animal Experimentation Committee for these experiments (permission no. ESAVI/7256/04.10.07/2014).

## Data Availability

Sampling metadata, morphological measurements, and radiation dosimetry data are available in Dryad (https://doi.org/10.5061/dryad.1zcrjdfrt).

## APPENDIX 1

### Internal dosimetry

To estimate internal dose rate caused by exposure to radio‐cesium contamination, ^137^Cs activity was measured by SAM 940 radionuclide identifier system (Berkeley Nucleonics Corporation) equipped with a 3" × 3" NaI detector. The detector was enclosed in 10‐cm‐thick lead shielding to reduce the noise from background radioactivity. The system was calibrated with reference standard sources. With corrections for laboratory background, the ^137^Cs activity of whole bodies was evaluated from the obtained spectra in the energies range from 619 to 707 keV (with cesium photopeak at 662 keV) with the use of the phantom with known activity and geometry similar to measured samples. Given the different time from capturing animals till their sacrificing, the initial ^137^Cs activity (at the trapping timepoint) was calculated with the model *A* = *be^λx^*, where *A* is calculated initial cesium activity; *b* is activity that was measured in animal bodies; *x* is time in hours (after trapping and before sacrificing); *e* is 2.72; and *λ* is elimination constant. The parameters of ^137^Cs elimination were found in our other study (Tukalenko et al., unpublished), and they were close to ones reported earlier (Gaschak et al., 2011).

For each measurement, the critical detectable level (decision threshold) was found as *Lc* *= k[Rb/Tb(1+Tb/Ts]^1/2^*, where *Lc* is critical level; *k* is 1.65 (coefficient, which determines 0.05 probability of type I error or false positive); *Rb* is counting rate of background; *Tb* is time of background measurement; and *Ts* is time of sample measurement (Isaev et al., 2010). The cesium activities above the critical level (decision threshold) were used for internal dose rate estimation; otherwise, the ^137^Cs activity was considered as zero.

With the use of conventional approach (Baltas et al., 2019; Cristy & Eckerman, 1987), an individual bank vole's internal absorbed dose from incorporated ^137^Cs acquired during one day was calculated (mGy/day) as a product of the activity of ^137^Cs incorporated in the animal's body (Bq/kg) and the unit conversion coefficient and the sum of all electron, positron, and photon energies emitted per decay of ^137^Cs and its daughter radionuclide ^137m^Ba. The energies were calculated taking into account the absorbed fractions for electron or positron or photon of the specific energy line, the intensity (or emission frequency) of the specific energy line (MeV) emitted per decay of ^137^Cs and its daughter radionuclide ^137m^Ba (ICRP, 1983), and the assumption that ^137^Cs source is uniformly distributed throughout homogeneous sphere of 20 g mass of unit density and tissue‐equivalent composition (Stabin & Konijnenberg, 2000). Despite the considerable contribution of ^90^Sr to the total internal absorbed dose, that is considered to be approximately the same as from cesium‐137 in Chernobyl (Beresford et al., 2020), in our study we neglected it because of characteristics of this beta‐emitting radionuclide (tissue‐specific strontium distribution in a body: ^90^Sr deposits mainly in bones and adds its adsorbed dose contribution there). The energies from alpha‐emitters (plutonium and americium: radioisotopes of transuranium elements) were neglected because of their low contribution to the total internal absorbed dose, less than 5% (Beresford et al., 2020).

Thus, the internal absorbed dose rates were as follows (median[interquartile range]): 0.0[0.0;0.0]*10^–3^ mGy/day at KYV‐CTRL in range of 0.0–1.5*10^–3^ mGy/day, 3.7[1.6;3.7]*10^–3^ mGy/day at CEZ‐CTRL in range of 1.6–3.7*10^–3^ mGy/day, and 1.4[0.4;3.7]*10^–1^ mGy/day at CEZ‐CNTM in range of 0.04–20.0*10^–1^ mGy/day.

As individual total absorbed dose rate, we considered the sum of estimations of internal and external absorbed dose rates per each animal. The total absorbed dose rates were as follows (median[interquartile range]): 5.1[3.6;7.2]*10^–3^ mGy/day at KYV‐CTRL in the range of 3.6–7.9*10^–3^ mGy/day, 9.5[6.0;10.3]*10^–3^ mGy/day at CEZ‐CTRL in the range of 3.6–11.1*10^–3^ mGy/day, and 5.3[4.2;9.3]*10^–1^ mGy/day at CEZ‐CNTM in the range of 2.7–34.1*10^–1^ mGy/day.

## APPENDIX 2

### Fluorescence in situ hybridization (FISH) to identify principal locations of Msat‐160 satellite in the bank vole genome.

Fibroblasts were isolated from male bank voles collected from contaminated area within the CEZ (Gluboke, mean external dose rate 21 μGy/hr) and from a control areas near Kyiv (see Mustonen et al. (2018) for cell culture conditions). Fibroblasts were treated with 0.01 µg/ml colcemid for 2 hr, after which the cells were trypsinized and resuspended to hypotonic 0.075 M prewarmed KCl. Cells were incubated for 20 min at 37°C, after which the cells were fixed in 3:1 methanol:glacial acetic acid according to the standard protocol (Franek et al., 2015).

For the chromosome preparations, 10 µl of the fixed sample was dropped on Superfrost slide and the slides were air‐dried. Slides were then quickly washed in 2× SCC and incubated for 1.5 hr in RNAse A (200 µg/ml, AppliChem) in 2× SSC. Slides were washed 3 times for 5 min in 2× SSC and incubated for 2 min in 0.01 M HCl, then for 5 min in pepsin (16 µg/ml in 100 mM HCl, 37°C), followed by 3 × 5 min wash in 2× SSC. Slides were incubated for 10 min in 4% formaldehyde in 2× SSC, washed for 3 × 5 min in 2× SSC, and dried in EtOH series (70%, 80%, and 96%). Biotin‐labeled PCR probes were hybridized on the slides overnight at 37°C. After the hybridization, slides were washed for 5 min in 2× SCC, then 3 × 5 min in 40% formamide/2× SCC (at 42°C), 1 × 5 min in 2× SCC at 42°C, 1 × 5 min in 2× SSC at RT, and 5 min in 4T buffer (4× SSC and 0.05% Tween‐20). 80 µl of blocking solution (4× SSC, 5% BSA, and 0.2% Tween‐20) was applied on the slides, incubated for 30 min at RT, and washed for 5 min in 4T buffer. For visualization of the Msat‐160 region, 80 µl of streptavidin‐AF594 (1:1,000 in blocking solution) was added to the slides and incubated for 30 min at 37°C, followed by 3 × 5 min washes in 4T, 1 × 5 min wash in 2× SSC, and dehydration in ethanol (70%, 80%, and 90%). Chromosomes were stained with DAPI (1 µg/ml in Vectashield, Vector Laboratories). Images were taken using Zeiss Axio Imager Z2 microscope, using a Plan‐Apochromat 100×/1.4 OIL objective and an ORCA Flash 4 camera. Images were analyzed with CellProfiler 2.4. (GFP for DAPI, Texas Red channel for Msat‐160).

The biotin‐dUTP‐labeled DNA probe for Msat‐160 was prepared in a 50 µl PCR with 0.25 mM of each dA/G/CTP, 0.05 mM of dTTP, 0.20 mM of biotin‐dUTP, 0.4 µM of both forward and reverse Msat‐160 primers (Table 1), 1.5 mM of MgCl_2_, 1X Taq Buffer with KCl, 2.5 U of Taq DNA polymerase (Thermo Scientific), and 10 µl of PCR product as a template. PCR program was as follows: 95°C for 3 min, and 30 cycles of 95°C for 10 s, 58°C for 5 s, and 72°C for 5 s, followed by elongation of 1 min at 72°C. MJ Thermal Cycler from Bio‐Rad was used. Template PCR product was produced with same conditions as above, except with 0.05 mM of dNTPs and 2.5 µl of DNA extracted from a bank vole liver sample (approximately 70 ng) as a template.
